# Protein Kinase Inhibitors as a New Target for Immune System Modulation and Brain Cancer Management

**DOI:** 10.3390/ijms232415693

**Published:** 2022-12-10

**Authors:** Alessia Filippone, Deborah Mannino, Giovanna Casili, Marika Lanza, Irene Paterniti, Salvatore Cuzzocrea, Anna Paola Capra, Lorenzo Colarossi, Dario Giuffrida, Sofia Paola Lombardo, Emanuela Esposito

**Affiliations:** 1 Department of Chemical, Biological, Pharmaceutical and Environmental Sciences, University of Messina, Viale Ferdinando Stagno D’Alcontres, 31, 98166 Messina, Italy; 2 Istituto Oncologico del Mediterraneo, Via Penninazzo, Viagrande, 7, 95029 Catania, Italy

**Keywords:** brain tumors, protein kinase inhibitor, immune system, tumor microenvironment, anti-tumor immunity

## Abstract

High-grade brain tumors are malignant tumors with poor survival and remain the most difficult tumors to treat. An important contributing factor to the development and progression of brain tumors is their ability to evade the immune system. Several immunotherapeutic strategies including vaccines and checkpoint inhibitors have been studied to improve the effectiveness of the immune system in destroying cancer cells. Recent studies have shown that kinase inhibitors, capable of inhibiting signal transduction cascades that affect cell proliferation, migration, and angiogenesis, have additional immunological effects. In this review, we explain the beneficial therapeutic effects of novel small-molecule kinase inhibitors and explore how, through different mechanisms, they increase the protective antitumor immune response in high-grade brain tumors.

## 1. Introduction 

Brain tumors are the most aggressive of the various types of cancer, caused by the uncontrolled and accelerated multiplication of cells in the brain. Around 75% of malignant brain tumors are classified as gliomas, which are neuroectodermal tumors arising from glial cells or precursors and include astrocytoma, oligodendrogliomas, and ependymomas [1]. According to the World Health Organization (WHO) central nervous system (CNS) tumor classification system, the glioma with the lowest survival rate and highest index of malignancy, designated as IV° grade, is glioblastoma (GBM) [2,3]. Despite multiple significant efforts and promising research results, there is a low improvement in the survival of patients with gliomas [4]. An important contributing factor to the development and progression of glioma is its ability to evade the immune system because of numerous mechanisms related to the interaction between tumor cells and immune cells which are involved in the immune evasion processes [5]. The lack of effector immune cell recruitment and the expansion of regulatory T cells further promote the immunosuppressive environment of gliomas [6]. The mechanisms of immune evasion mainly contribute to the progression of brain tumors by directing the tumor cells towards a malignant neoplastic phenotype with high metastasizing power. On the other hand, the mechanisms that could trigger carcinogenesis processes and promote the uncontrolled cell proliferation of cancer cells are related to the dysregulation of the protein kinase signaling [7].

Protein kinases through a cascade signaling network by phosphorylating surface receptors and cytoplasmic enzymes can activate different mechanisms involved in cell proliferation, differentiation, and angiogenesis, promoting tumor development. [8]. Genetic alterations that cause protein kinase overexpression are involved in the oncogenesis of various types of cancer so become highly attractive pharmaceutical targets leading to the development of numerous kinase inhibitors (KIs) [9]. In support of these notions, numerous preclinical studies mentioned in this review showed that KIs are capable of increasing anti-tumor immunity suggesting a dual therapeutic effect in counteracting the progression of brain tumors. Since future research should hope to reduce immunosuppressive mechanisms to generate more beneficial treatment options, this review aimed to discuss new therapeutic strategies targeting protein kinases that are simultaneously capable of increasing anti-tumor immunity in the context of brain tumors.

## 2. Basis on the Existing Relationship between the Immune System and Varied Brain Tumors 

**Figure 1:**
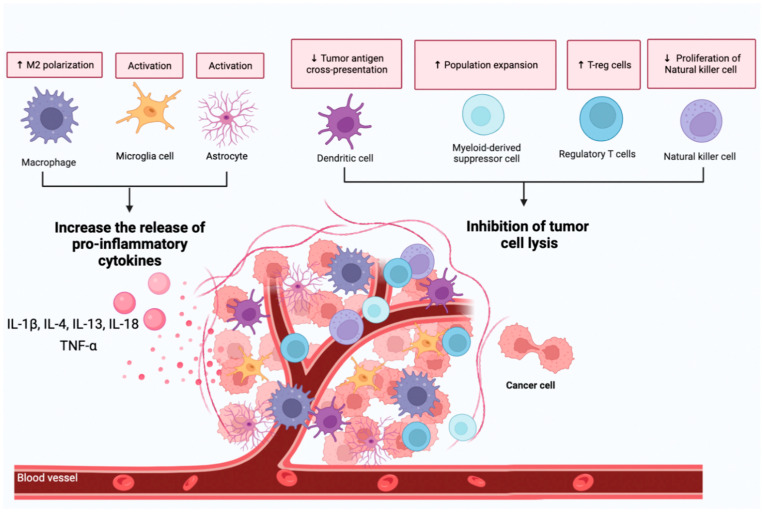
Immunosuppression in brain cancer. The increase of M2 macrophages, activation of microglia and astrogliosis promote a chronic systemic inflammatory environment associated with tumor development and metastasis. In addition, the evasion of the immune system by cancer cells creates an immunosuppressive environment that contributes to tumor growth. The mechanisms of immunosuppression are related to a reduction of the cytotoxic activity of dendritic cells and NK cells and to the increase of cells capable of reducing the activity of CD8+ T-cells such as T-reg and MDSC cells. The figure was created with the help of Biorender.com (accessed on 10 May 2022).

The most noticeable quality of CNS and the immune system is the capacity to communicate signals to far-off parts of the body with exceptional particularity and variety. A prominent presence of immune cells in the brain consists of microglia, which include 80% of brain immune cells in combination with different immune cells types such as myeloid cells, monocytes/macrophages, DCs, T cells B cells, and natural killer (NK) cells that participate in complex brain physiological and pathological processes [10]. In the steady state, immune cells such as astrocytes and microglia support neurons, modulate neuronal excitability and plasticity, directly control neurotransmitter uptake, and regulate angiogenesis/vascularization [11]. During pathological conditions including traumas, neurodegenerative disorders, and also brain tumors, resident immune cells in the brain can create a barrier that avoids inflammatory cells entry potentiating the brain’s response to injury. However, the exacerbation of the pathological state over time throughout the grey and white matter in the CNS triggers dynamic activation of microglia, astrocytes, and resident macrophages that release cytokines such as interleukins (ILs) (IL-1β, IL-4, IL-13, IL-18), tumor necrosis factor α (TNF-a), and chemokines (CX3CL1) that extend inflammatory state in CNS (Figure 1) [12]. Indeed, a cross-talk between tumor cells and the immune system exists and is played exactly by resident immune cells in the brain, mainly involved in the progression of gliomas. Genetic alterations of ligands or receptors in CNS are involved in brain tumor progression and the suppression of the immune state through them may be considered a privileged approach. For example, the human leucocyte antigen (HLA) system, also known as the major histocompatibility complex (MHC) may exhibit immunosuppressive actions in various types of tumors by reducing malignant glioma cells elimination by NK cells and increasing the growth of glioma cells [13,14]. Also, it has reported that immunological tumor features were associated with isocitrate dehydrogenase (IDH) mutational status. Low-grade gliomas (LGGs) and GBM have shown IDH 1/2 mutations may be due to specific effects of it on tumor-associated immune response with genetic abnormalities in tumor protein 53 (TP53) gene [15]-encoding p53 protein, which modulates cell cycle progression and apoptosis, and functions as a tumor suppressor [16]. In addition, the IDH1/2 genetic alterations can be also associated with abnormal expression of platelet-derived growth factor (PDGF), leading to the abnormal activation of microglia and promoting the tumor cell’s invasiveness [17].

## 3. Protein Kinase Involvement as the Main Players in the Progression and Development of Malignant Brain Tumors: Overview on Astrocytoma and Glioblastoma

There are various cellular mechanisms and signaling pathways that enable cells to grow, proliferate, differentiate, and carry out all physiological processes and are required for survival and/or apoptosis. Under normal physiologic conditions, one of these mechanisms is represented by the coordinated action of specific kinases and phosphatases capable of adding and removing phosphate groups respectively to control the activation state of certain proteins. More than 518 kinases [18] and 156 phosphatases [19] have been discovered in the human genome which play important roles in cell functionality. Despite the intense scientific research in this area, it is known that the abnormal activation of protein phosphorylation driven by numerous kinases often represents a direct consequence of many characteristic phenotypes of tumor biology such as proliferation, survival, motility, metabolism, angiogenesis, and evasion of antitumor immune responses [20]. In the brain cancer, numerous genetic alterations such as gain-of-function mutations, genomic amplification, chromosomal rearrangements, and/or autocrine activation promote the anomalous oncogenic activation of protein kinases which causes an increase in the activity of the kinase itself. In particular, tyrosine kinase receptors (RTK) are more frequently overexpressed as a consequence of gene amplification. In brain tumors, the activation of the signaling pathways of protein kinases increases the processes of cell proliferation, survival, and differentiation that lead to the initiation and maintenance of the tumor [21].

## 4. Strategies for Targeting Malignant Brain Tumors with Kinase Inhibitors: VEGF, HER2, CSF-1R, PI3K, ERKI/II Targeting

**Figure 2:**
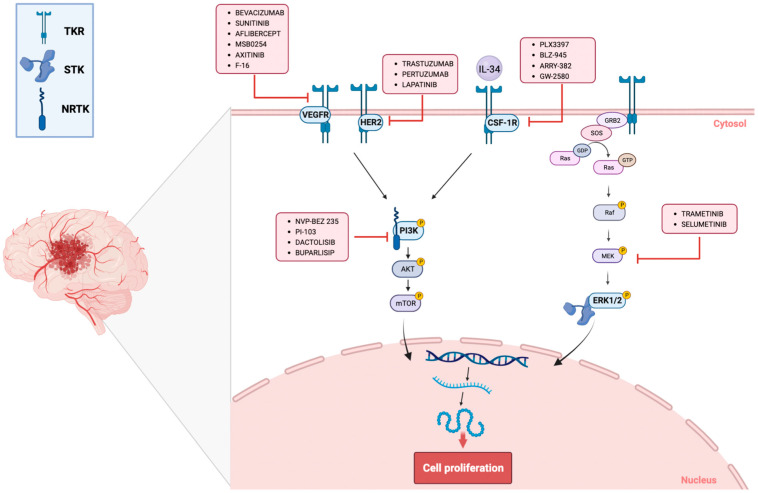
Effects of kinase inhibitors drugs in brain tumors. In brain tumors, inhibition of kinases such as TKR, STK, and NRTK by new molecules significantly reduced the proliferation, migration, and invasion of cancer cells in clinical and preclinical studies. The figure was created with the help of Biorender.com (accessed on 30 May 2022).

The discovery that protein kinases, through phosphorylation of surface receptors and cytoplasmic enzymes, could potentially trigger carcinogenesis, led to major efforts to develop selective inhibitors useful for the treatment of a wide range of brain tumors (Figure 2) [22]. KIs represent an important and still emerging class of targeted therapeutic agents. Today, more than 25 oncology drugs that target kinases have been approved, while numerous additional therapeutics are in various stages of clinical evaluation [23,24]. KIs were developed as a group of small molecules that can cross cell membranes and interact with different binding sites of protein kinases. Preventing protein kinase phosphorylation leads to the blocking of various downstream signaling cascades. The mechanism of action of KIs can change according to the activation state of the kinase protein which is preferentially recognized (active or inactive), to the binding mechanism (reversible or covalent), and to the binding site (adenosine triphosphate named ATP, or allosteric binding pocket) [25]. Molecules that target the ATP-binding pocket when the enzyme is in its active conformation are classified as class I KIs; type II KIs recognize and maintain the inactive conformation of the enzyme kinase; class III KIs block enzyme activity by interacting with a pocket adjacent to the ATP binding site in a mechanism that does not compete with ATP binding; class IV KIs bind to a different allosteric binding from substrate/ATP binding sites. Other classes of KIs include type V inhibitors which, by reacting with a nucleophilic cysteine residue, can form irreversible covalent bonds with the active site of the kinase, typically allosteric small-molecule KIs [26]. Targeted kinases include tyrosine kinases (TKs) and serine/threonine protein kinases (STKs). TKs are divided into two classes, non-receptor tyrosine kinases (NRTKs) and receptor tyrosine kinases (RTKs). NRTKs are cytoplasmic proteins that play important roles in cell signaling and regulation of gene expression; they can be bound to the cell membrane or be specific to the nucleus. RTKs are transmembrane proteins that have phosphorylating activity and are selective towards tyrosine residues [27]. RTKs are involved in mediating cell-to-cell communication and controlling biological functions, including cell growth, motility, differentiation, and metabolism [28]. Comprehensive genomic analysis of multiple tumor types, including low-grade and high-grade gliomas, has identified the acquisition of somatic genetic alterations in RTKs as one of the most common sources of aberrant intracellular signaling. Moreover, constitutive activation of RTK signaling confers oncogenic properties to normal cells triggering the carcinogenesis process [29]. STKs catalyze the phosphorylation of proteins on some of their serine or threonine residues. They play a vital role in regulating cell survival signals in response to growth factors or cytokine stimulation. Alterations of these kinases have been associated with tumor growth, metastasis, and poor clinical outcome. The most affected signaling pathways in glioma cells involve RTKs and their downstream pathways, such as the phosphatidylinositol 3-kinases (PI3K/AKT/mTOR) and mitogen-activated protein kinase pathways (MAPK) [30].

### 4.1. Angiogenic Inhibition in High-Grade Gliomas by Targeting VEGFR

Vascular endothelial growth factor receptor (VEGFR) is an RTK involved in glioma growth. There are three VEGF receptors, VEGFR-1, -2, and -3. Physiologically, VEGFR-1 and VEGFR-2 are found in the membranes of vascular endothelial cells and are the central regulators of vasculogenesis (the formation ex Novo of the circulatory system) and angiogenesis (formation of new vessels), VEGFR-3 is found mainly on lymphatic endothelial cells and regulates the lymphangiogenesis (formation of lymphatic vessels from pre-existing lymphatic vessels). To date, five physiological ligands for VEGFR have been identified in mammals (VEGF-A, VEGF-B, VEG-C, VEGF-D, and placenta growth factor or PIGF) [31]. VEGFR was found to be overexpressed in nearly all types of human cancer, especially in the hypoxic regions of tumors and in blood vessels in/near tumors. In brain tumors, such as GBM and astrocytoma, binding of VEGF to VEGFR on tumor blood vessels greatly improves permeability and activates endothelial cell proliferation, survival, and migration by promoting tumor growth. For these reasons, suppressing angiogenesis by blocking the VEGF/VEGFR signaling axis could be a therapeutic strategy in brain tumor treatment [32]. VEGFR suppression can be achieved by targeting the ligand or by directly targeting the receptor. Over time, numerous molecules have been discovered that can inhibit VEGFR. Sunitinib, a small molecule that inhibits VEGF/VEGFR signaling through the selective inhibition of multiple tyrosine kinase receptors such as VEGFR-1,2,3, PGDFR-α, and β is undergoing phase two clinical trials in glioma patients [33]. In vivo studies demonstrated a synergistic effect of Sunitinib with temozolomide (TMZ) by showing a significant reduction in tumor growth compared with mono treatment with TMZ [34]. Another drug undergoing phase II of clinical trials in glioma patients is Bevacizumab, a recombinant humanized monoclonal antibody. Bevacizumab is approved for use in recurrent GBM in the USA, Canada, and many other countries outside the European Union (EU) [35]. The drug binds all the VEGF isoforms with high affinity, thus preventing the binding and consequent activation of VEGFR. This could prevent the growth of new blood vessels and inhibit tumor growth [36]. Additionally, it was well tolerated and studies noted an anti-edema effect of this drug, allowing a reduction in the use of corticosteroids in about a third of patients [37]. Preliminary data from phase II clinical trials in patients with recurrent malignant glioma demonstrated the efficacy of Trap (aflibercept), a molecule synthesized using the recombinant DNA technique. Trap (aflibercept) targets VEGFR ligands by sequestering all VEGF and PlGF isoforms and improving the survival of GBM patients [38]. The effect of another anti-VEGFR2 monoclonal antibody, MSB0254, on the angiogenesis of GBM cells has recently evaluated in preclinical studies. The pharmacological effects of GBM remain mysterious. Recent results have demonstrated that MSB0254 significantly reduced tumorigenicity and the formation of vascular mimicry in both in vitro and in vivo models of GBM, but this agent remains far away of being a successful oncology drug candidate [39]. Furthermore, Axitinib (AG-013736), a selective inhibitor of VEGFR1-3, PDGFR, and c-KIT, showed good single-agent efficacy with a manageable toxicity profile in patients with recurrent GBM [40]. It has been suggested that Axitinib treatment had a favorable impact on immune function in patients with recurrent GBM [41]. In this context, we would mention a further pre-clinical evaluation that studied the effects of a new small molecule named F16 having selective inhibitory activity for VEGFR. Pharmacokinetic studies have shown that F16 can cross the blood–brain barrier without showing neurotoxic effects. Moreover, a group of researchers has recently studied the effect of F16 in an in vitro and in vivo model of GBM, showing that inhibition of VEGFR by F16 delayed tumor growth by reducing angiogenesis and reduced the processes of migration and invasion [42].

### 4.2. Pharmacological Inhibition of HER2 in High-Grade Gliomas

In addition to being involved in the initial processes of carcinogenesis, the receptors with tyrosine kinase activity play a fundamental role in the more advanced stages of cancer. Human Epidermal Growth Factor Receptor 2 (HER2) is a 185 kDa transmembrane RTK, physiologically involved in the signal transduction processes that lead to cell growth and differentiation [43]. In humans, mutations that increase HER-2 expression have identified in 20%–30% of breast cancers. Numerous studies have highlighted the association between the amplification of HER2 and an increased risk of CNS metastasis in 20% of women who carry a primary diagnosis of breast cancer [44]. The phosphorylated tyrosine binds to intracellular signaling molecules leading to the transcription of genes involved in cell proliferation, survival, differentiation, and angiogenesis [45]. Thanks to its oncogenic power, HER-2 has become the target of numerous new-generation therapies [46]. Trastuzumab was the first FDA-approved drug targeting HER2. It is a humanized monoclonal antibody that binds the extracellular domain of the receptor preventing its homo- or heterodimerization and therefore its activation [47]. The main limitation of this drug is that it has a high molecular weight and does not penetrate the blood–brain barrier, even in the presence of brain metastases. To overcome this limit, a clinical study evaluated the efficacy of intrathecal administration of Trastuzumab in patients with HER2-positive leptomeningeal metastases. The results demonstrated that intrathecal Trastuzumab administration could be a viable option for HER-positive patients [48,49]. A meta-analysis of 12 phases II clinical trials demonstrated the beneficial effect of Lapatinib, a double epidermal growth factor receptor (EGFR) and HER2 inhibitor, in patients with progressive HER2-positive CNS metastases. It has a greater ability to cross the BBB, and in combination with capecitabine, a prodrug of 5-fluorouracil (5-FU), significantly reduced the number of patients experiencing CNS progression [50]. Molecules of natural origin have also been studied. For example, a recent study showed potent inhibitory effects against EGFR and HER2 kinases of some derivatives of Cytisine N-methylene-(4′,7-dihydroxy-3′-methoxy)-isoflavone. This new class of EGFR/HER2 inhibitors paves the way for further research in tumors with metastases to the nervous system field [51].

### 4.3. CSF-1R Kinase Inhibitors Reduce Glioma Progression through TAMs Depletion

The glioma microenvironment is dominated by macrophages and microglia, collectively known as tumor-associated macrophages (TAMs) that contribute to the progression and maintenance of cancer [52]. Some receptors such as the colony-stimulating growth factor receptor-1 (CSF-1R) can activate these macro-phages and constitute new targets for glioma. CSF-1R is expressed in myeloid cells such as macrophages, dendritic cells (DCs), neutrophils, and myeloid-derived suppressors (MDSCs) within the tumor microenvironment (TME). In the TME, CSF1 induces the homodimerization of the receptor and the subsequent activation of proliferation, differentiation, and survival of monocytes and macrophages increasing the cytotoxicity of tumor cells. In brain tumors such as GBM, overexpression of CSF-1R TME has demonstrated to promote the recruitment of TAMs [53,54]. Therefore, new molecules capable of inhibiting CSF-1 or its CSF-1R receptor in intratumoral macrophages are another potential therapeutic strategy for GBM [55]. Among CSF1R inhibitors used in clinical trials, there is Pexidartinib (PLX3397). In addition to inhibiting CSF-1R, it inhibits c-KIT, the mutant fms-like tyrosine kinase 3 (FLT3), and the platelet-derived growth factor receptor (PDGFR)-β. It is used in classical Hodgkin’s lymphoma (cHL), neurofibromas, sarcomas, and leukemias. An in vitro study using normal microglia cells obtained from C57Bl/6J mice and GL261 murine GBM examined the effect of the invasion of GBM microglia by a matrigel-coated invasion chamber. The study showed that inhibition of CSF-1R signaling using PLX3397 reduced the number of microglia/macrophages and significantly inhibited invasiveness. Furthermore, in vivo studies demonstrated that this compound readily crosses the normal BBB and reaches the CNS without being actively transported [56]. Small molecules targeting CSF1R such as BLZ945, ARRY-328, and GW2580 are being studied. BLZ-945 is a small molecule inhibitor of CSF-1R whose safety and efficacy have been evaluated in phase I/II clinical trials in patients with a variety of advanced solid tumor types [57]. The use of this molecule in brain tumors such as GBM has recently been studied in a mouse model of glioma. In this study, the researchers wanted to examine whether BLZ-945 could enhance the beneficial effects of radiotherapy in GBM. The results showed that this combination increased RT-induced antitumor immunity, reduced anti T inflammatory/immunosuppressive macrophage phenotype polarization (M2) within the TME, and reduced tumor growth rates. RT and BLZ-945 combination represent a promising therapeutic strategy to be investigated clinically in GBM. A further study confirmed these results in an in vitro and in vivo model of GBM. The in vitro model demonstrated that the inhibition of CFS-1R in polarized M2 macrophages did not change the total number of macrophages but significantly reduced their M2 polarization. The in vivo model, an orthotopic immuno-competent GBM model, showed that BLZ-945 alone did not improve median overall survival compared to control mice, whereas the combination of BLZ-945 with radiotherapy improved survival and therapeutic response in GBM [58]. ARRY-382 and GW-2580, innovative highly selective inhibitors of CSF1R, have demonstrated good tolerance and safety in clinical phase studies in patients with advanced or metastatic tumors but their effect in brain tumors such as gliomas has not yet been evaluated [59,60]. 

### 4.4. Activity of Novel PI3K Inhibitors in Vivo and in Vitro Glioma Models

Since targeting therapy against the TME is an excellent opportunity to reduce GBM progression, an additional protein kinase involved in the CFS-1R pathway that could constitute a new therapeutic target is a phosphatidyl-inositol-3 kinase (PI3K). The PI3K family consists of different isoforms: PI3K-α, PI3K-β, and PI3K-γ. The activation of CFS-1R induces cascade phosphorylation processes, leading to the activation of PI3K-γ in myeloid cells, which promotes the infiltration of TAMs M2 and the increase of the tumor suppressive immune microenvironment [61,62,63]. Today, few studies have evaluated the double inhibition of PI3K and CSF-1R. An in vitro and in vivo study in pancreatic cancer models demonstrated that double inhibition by using nano micelles containing NVP-BEZ 235 (PI3K-γ inhibitor) and CSF-1R-siRNA (CSF-1R inhibitor) remodeled the TAMs and synergistically activated antitumor immune responses, providing an alternative approach in counteracting tumor progression [64]. In tumor cells, PI3K is activated by several growth factors such as epidermal growth factor receptor (EGFR) and platelet-derived growth factor receptor (PDGFR). Through protein kinase B (AKT), PI3K participates in the phosphorylation and consequent activation of mammalian targets of the rapamycin (mTOR) complex. The PI3K/AKT/mTOR pathway is hyperactivated in several types of astrocytic tumors, mostly in GBM where it stimulates cell survival and cell growth and guides the progression of malignant glioma. Recently, a new KI, PI-103, which inhibits both PI3K- α (a main isoform driving malignant progression in glioma) and mTOR signaling, was used in several preclinical glioma models. PI-103 can reduce the expression of the anti-apoptotic protein p-AKT and significantly reduce the proliferation of U-373 human GBM cells. Furthermore, the combinatorial inhibition of PI3K-α and mTOR was well tolerated models of glioma [65]. One study demonstrated the beneficial effects of dactolisib, a PI3K inhibitor, in patient-derived GBM cells. The inhibition significantly reduced tumor cell proliferation and increased apoptosis processes [66]. Another ideal candidate for therapeutic strategies targeting PI3K isoforms is buparlisib. Pharmacokinetic studies demonstrated that buparlisib accumulates in the brain and inhibits PI3K-isoforms at clinically achievable plasma concentrations. For this reason, it is currently in clinical development for various cancers, including primary and secondary brain tumors [67].

### 4.5. ERK Inhibition Suppresses Glioma Growth

Extracellular signal-regulated kinases (ERK) belong to STKs proteins. ERK1 and ERK2, (44 and 42 kDa) are two important members of the RAF-MEK1/2-ERK1/2 RAS-regulated pathway, which act downstream of this signal. ERK1/2, are generally found in the cytoplasm, and are activated when growth factors, cytokines, viruses, G protein-coupled receptor ligands, or oncogenes promote dimerization and activation of rapidly accelerated fibrosarcoma (RAF) kinases. RAF kinases proteins phosphorylate and activate mitogen-activated protein kinase1/2 (MEK1/2), which phosphorylates and activates ERK1/2. Once activated, ERK is translocated to the nucleus to regulate the activity transcription factors such as c-Myc, which is involved in cell cycle progression and other fundamental cellular processes involved in cell differentiation [68]. This signaling pathway has attracted particular attention because it is deregulated in a variety of tumors such as GBM and astrocytoma [69]. This has led to the development of pharmacological inhibitors capable of blocking RAF-MEK1/2-ERK1/2 signaling in cancer. The use of RAF or MEK inhibitors such as Trametinib has led to resistance phenomena involving mechanisms such as KRAS or BRAF amplification, and MEK mutation with consequent ERK1/2 reactivation. Therefore, today a greater interest has arisen in targeting the path to the level of ERK1/2 to reduce the adverse effects of inhibitors already used in clinical studies. In recent years, many inhibitors of ERK1/2 activity have been described in the literature, some of which have progressed in clinical trials. Most of the developing ERK inhibitors are reversible and ATP competitive. They can block the phosphorylation activity of the catalytic site of ERK1/2 or they can prevent the formation of the active conformation of ERK1/2 [70,71,72]. Recently, a study investigated the effects of Selumetinib, an ERK1/2 inhibitor, loaded in exosomes derived from human U87 GBM cells in an in vitro and in vivo model of GBM. It has observed that the tropism exhibited by GBM-derived exosomes can be used to transport Selumetinib to tumor sites. Furthermore, these Selumetinib-loaded GBM-derived exosomes had a specific antitumor effect on U87 GBM cells and were non-toxic to normal brain cells, offering better therapeutic prospects for GBM therapy [73].

## 5. Impact of Kinase Inhibitors on Immune System

**Figure 3:**
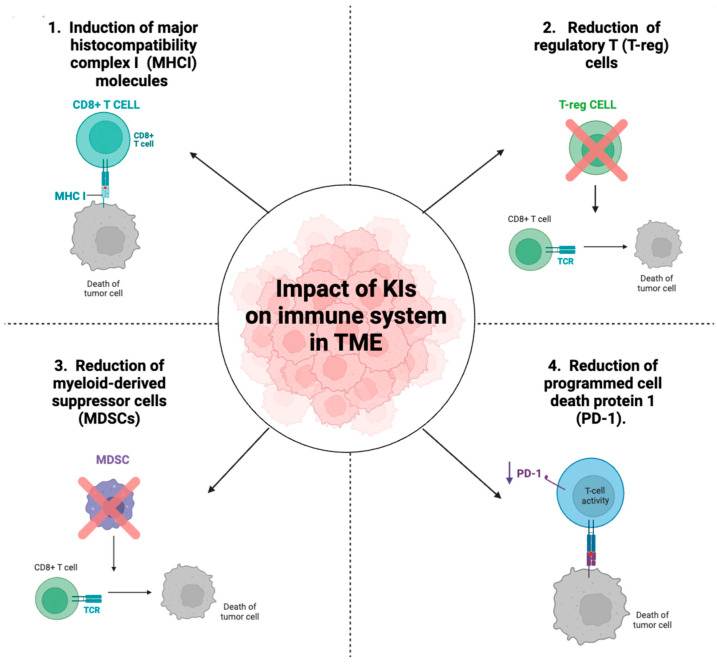
Impact of KIs on immune cells in TME. Some KIs through intrinsic mechanisms linked to the blocking of protein kinases, in addition to reducing the growth of cancer cells, can induce anti-tumor immunity. Some KIs mediated anti-tumor immunity through several mechanisms. These include: induction of MCHI molecules which increases the cytotoxic activity of CD8+ T-cells (panel 1), reduction of T-reg (panel 2), and MDSC cells (panel 3), causing an increase in immune cells and a reduction of PD1 expression on lymphocytes, which increases their cytotoxic activity (panel 4). The figure was created with the help of Biorender.com (accessed on 6 September 2022).

Recent studies have shown that KIs, in addition to their canonical function in modulating the processes of cell proliferation and angiogenesis in tumors, have additional immunological effects. Since immunosuppressive mechanisms are fundamental for cancer cell growth, immune cells have become compelling targets for anticancer therapy, so, a better understanding of the possible effects of KIs in the context of tumor immunity is a key goal in the oncology field today. The molecular mechanism by which KIs modulate immune system is still not clear today but some studies show that KIs can contribute to a protective antitumor immune response by reducing the number and activity of various types of immune cells involved in the evasion mechanisms of the immune system in brain cancer such as regulatory T cells (T-reg), myeloid-derived suppressor cells (MDSC), and programmed cell death protein 1 (PD-1) reduction (Figure 3). T-reg cells (CD3+ CD4+ CD25 and FoxP3+), known as potent suppressors of the adaptive immune system, inhibit the activity of cytotoxic T cells (CD8+ T cells) and helper T cells (CD4+ T cells). Accumulation of T-reg in patients with GBM correlates with a poor prognosis and a higher degree of malignancy [74]. Furthermore, MDSCs are also cells that are abundantly recruited and expanded in the TME of patients with GBM leading to immunosuppression [75]. Numerous mechanisms have been reported by which MDSCs inhibit immune responses, including inhibition of the antitumor activity of CD8+ T cells, suppression of natural killer (NK) cells, macrophage and DC function, and induction of T-regs [76]. Based on these considerations, MDSC and T-reg cells are central immunosuppressive cells involved in the progression of brain tumors. Furthermore, PD-L1 expression in glioma cells has also shown great variance in several studies that attribute a therapeutic and prognostic biomarker role to PD-L1 in glioma [77]. PD-L1 expressed by glioma, in addition to increasing the number of T-reg cells, inhibits the functions of T lymphocytes and induces apoptosis of tumor-specific CD4+ and CD8+ T lymphocytes by decreasing the production of cytokines such as Inter-leukin-2 (IL-2) and interleukin-10 (IL-10) [78,79]. The discovery that some KIs may have immunological effects emerged when studies showed that RTK inhibitors increased the number of mature DCs. In cancer patients, the frequency of DC, the antigen presenting cells that physiologically induce the antitumor immune response, is significantly lower than in healthy individuals [80]. Subsequently, researchers began studying further molecular mechanisms by which KI could increase antitumor immunity, and new discoveries were made. Sunitinib, a VEGFR inhibitor previously mentioned because it is undergoing in clinical trials in patients with GBM, modulated the TME in the context of the immune system. Treatment with Sunitinib reduced the immune suppressive function of MDSCs but also prevented the development of Treg. The number of MDSCs and T-regs (Foxp3+CD25+CD4+ cells) isolated from the spleen of Sunitinib-treated tumor-bearing mice was reduced in a dose-dependent manner. These results suggested that by decreasing T-reg and MDSC numbers, Sunitinib may modulate the TME. In addition, researchers identified the specific subsets of leukocytes and costimulatory molecules expressed in the tumors of sunitinib-treated or control mice by flow cytometry. Results demonstrated that there was a significantly higher percentage and infiltration of CD8+ and CD4+ cells in the tumors of the sunitinib-treated mice than in the control mice. In contrast, a significant reduction of PDL-1 was observed in the treated mice compared with untreated mice [81]. In another study B. Raychaudhuri et al. [82] investigated whether sunitinib had an effect on modulating tumor-associated immunosuppression. This study showed that in the mouse model of GBM, treatment with Sunitinib reduced the number of MDSCs which coincided with an increase in CD4+ cells. These data suggest that sunitinib can induce anti-tumor immunity by reduction of MDSC and T-reg cells and increase of CD4 and CD8 cells, thus improving the efficacy of immune-based cancer therapy for advanced malignancies. Although many molecular mechanisms are still to be elucidated, some studies observed that VEGFR inhibitors such as Sunitinib induce programmed cell death of MDSCs and T-reg cells thus exerting a direct effect on immune cells. Differently, other studies suggested that the effect of KIs on immune cells is closely related to the inhibition of the kinases themselves and indirectly also affects immune cells. For example, VEGFR1 inhibitors through anti-angiogenic activity can normalize tumor microvasculature and reduce the infiltration of MDSCS and T-reg lymphocytes [83]. A clinical study investigated the effects of axitinib and axitinib plus lomustine on immune function in patients with recurrent GBM. Axitinib, an inhibitor of VEGFR-1, 2, and 3, increased the number of naïve CD8+ T cells and central memory CD4+ and CD8+ T cells and reduced T-reg expression, leading to better antitumor immune responses in patients with recurrent GBM [41]. Another key process that regulates antitumor immunity is the presentation of peptide antigens by major histocompatibility complex class I (MHCI) and class II (MHCII) molecules. Tumors often downregulate the expression of MHCI and/or II molecules; indeed, a loss of MHC expression is associated with a poor prognosis and/or a worse response to immunotherapy. Studies have shown that PI3K, through the AKT pathway, represses the activity of MHCI and MHCII both. Given the involvement of PI3K in antitumor immunity, the researchers wanted to study the possible effects of Dactolisib, a PI3K inhibitor used for the treatment of GBM, on the immune system. Data showed that Dactolisib increased the expression of MHCI and MHCII molecules on epithelial cells enhancing antigen presentation [84]. The intrinsic expression of PD-L1 is associated with the PI3K/Akt/mTOR signal, which is why buparlisib-mediated inhibition of this pathway was under further investigation. In head and neck squamous cell carcinoma, treatment with buparlisib downregulated PD-L1 in total cell lysates. These results implied that the blockage of the PI3K/Akt/mTOR pathway could be a good adjunct therapy for patients showing poor response to therapy immune checkpoint [85]. Another PI3K inhibitor, IPI-549, reduced the suppressive activity of murine and human MDSCs cells and T-lymphocytes in an in vitro study [86]. Here, gene and protein expression analysis of whole tumor tissue collected from mice treated with IPI-549 revealed a higher frequency of circulating tumor-specific T cells, a higher percentage of tumor-infiltrating CD8+, IFNγ, T cells and a reduced percentage of T-reg cells, leading to an increase in the ratio of CD8+/T-reg cells. These results indicate that IPI-549 increases antitumor immunity by remodeling the immune microenvironment. Furthermore, IPI-549 is currently under development, both as a single agent and in combination with an anti-PD-1 antibody in solid tumors [87]. Regarding GBM, a study investigated the combinatorial effect of IPI-549 and an anti-PD-L1 antibody in an in vivo model using TMZ-resistant murine glioma-initiating cells. These data showed that treatment with anti-PD-L1 antibodies significantly reduced the infiltration of M2 into tumors, while the combined therapy of PD-L1 antibodies and IPI-549 significantly inhibited tumor growth [88]. Many anti-cancer drugs induce the tolerogenic death of cancer cells. This type of cell death lacks the help of T lymphocytes and is mediated by dendritic cells that engulf dying cancer cells and induce the production of immunosuppressive factors that promote cancer growth and invasiveness. In contrast, the scientific research mentioned in this review showed that many KIs invoke an immunogenic-type tumor cell death through different mechanisms such as the increase of CD8+ T-lymphocytes. Although the numerous pharmacological effects of many KIs still need to be investigated, the new data on their immunogenic antitumor activity mean that they can be exploited for superior clinical efficacy.

## 6. Side Effects and Resistance Phenomena of Current KIs

KIs are highly effective in many types of cancer, but often develop adverse events that can reduce therapy compliance. Toxicities from small-molecule KIs are related to the inhibitory effect of kinases in areas of the body other than those of cancer. For this reason, Structure–Activity Relationship (SAR) studies of drugs are still needed today to improve the selectivity of these inhibitors. The inhibition of kinases in organs where they play important physiological roles causes a series of toxic effects that lead the patient to use additional drugs. Heart RTK blockage causes the activation of the intrinsic apoptotic pathway and depletion of ATP in cardiomyocytes, resulting in left ventricular dysfunction, heart failure, and hypertension [89].

For these reasons, the side effects of Axitinib, a VEGFR inhibitor, occur in over 30% of patients and include diarrhea, hypertension, fatigue, loss of appetite, nausea, decreased kidney function, and anemia [90,91]. Since VEGFR is involved in the continuous renewal of capillaries, the blocking of these receptors could prevent the physiological repair of traumatized capillaries, facilitating the onset of bleeding. The RTKs, the target of many KIs, are also involved in maintaining the integrity of the mucous membranes and are a powerful mitogen of the gastric epithelium, so their inhibition justifies the observed gastrointestinal events. Another aspect that limits the use of KIs is the onset of resistance phenomena [92]. The most frequent cause is related to the activation of parallel signaling pathways that bypass the inhibited protein kinase. For example, the use of VEGFR inhibitors was observed to cause activation of all three members of the PI3K/AKT/mTOR pathway in subjects with GBM. Activation of the PI3K pathway has been associated with reduced levels of apoptosis and a reduction in survival times. Furthermore, genetic mutations of target kinases that promote reduced drug affinity are among the causes of resistance phenomena. Otherwise, genetic mutations promote the amplification of target kinases that reduce the pharmacological effect of KIs [93]. In conclusion, given the dual clinical utility of KIs in suppressing the tumor and increasing antitumor immunity, the clarification of the mechanisms of resistance should continue to be pursued.

## 7. Conclusions

The discoveries on the immunological properties of KIs continue to improve the obtained results during the pharmacological treatments GBM-patients. One of the main objectives still to be achieved is to understand the chemical–biological mechanisms regarding the immunomodulatory effect mediated by many KIs to make the most of this new class of drugs. Furthermore, it is important to consider the pharmacokinetic characteristics of KIs for the treatment of CNS tumors in order to evaluate their ability to cross the BBB. The ability of a drug to cross the BBB is closely related to its physicochemical properties, its polar surface area, its molecular weight, and its ability to act as a substrate for the efflux transporter of P-glycoprotein. Pharmacokinetic studies showed that decreasing key properties such as PSA to enhance BBB penetrance could produce a better substrate for cytochrome P4502D6, an undesirable effect as the polymorphic nature of this drug-metabolizing enzyme causes differences in metabolism between individuals. Therefore, also considering the physicochemical characteristics of KIs in the early stages of drug design represents a promising strategy for the use of these drugs in the treatment of brain tumors.

## Data Availability

Not applicable.

## Abbreviations

CNS: central nervous system; GBM: glioblastoma; WHO: World Health Organization; KI: kinase inhibitors; DCs: dendritic cells; MDSCs: myeloid-derived suppressors; TAMs: tumor-associated macrophages; TME: tumor microenvironment; HLA: human leucocyte antigen; MHC: major histocompatibility complex; PDGF: platelet-derived growth factor; T-reg cells: regulatory T cells; PD-1: programmed cell death protein 1; NK: natural killer TKs: tyrosine kinases; STKs: serine/threonine protein kinases; NRTKs: non-receptor tyrosine kinases; RTKs: receptor tyrosine kinases; PI3K: phosphatidylinositol 3-kinases; MAPK: mitogen-activated protein kinase; VEGFR: Vascular endothelial growth factor receptor; EGFR: epidermal growth factor receptor; HER2: Human Epidermal Growth Factor Receptor 2; CSF-1R: colony-stimulating growth factor receptor-1 mTOR: mammalian targets of the rapamycin; ILs: interleukins; TNF- α: tumor necrosis factor α; SAR: Structure-Activity Relationship.
